# The dual functions of the pentacyclic triterpenoid madecassic acid in ameliorating doxorubicin-induced cardiotoxicity and enhancing the antitumor efficacy of doxorubicin

**DOI:** 10.7150/ijbs.97418

**Published:** 2024-10-07

**Authors:** Wenlin Li, Kun Xu, Ming Lan, Junpeng Gao, Lin Dou, Yao Yang, Que Wang, Mingjing Yan, Sainan Li, Qinan Ma, Weimeng Tian, Beidong Chen, Ju Cui, Xiyue Zhang, Jianping Cai, Hua Wang, Liang Sun, Jian Li, Xiuqing Huang, Tao Shen

**Affiliations:** 1 The Key Laboratory of Geriatrics, Beijing Institute of Geriatrics, Institute of Geriatric Medicine, Chinese Academy of Medical Sciences, Beijing Hospital/National Center of Gerontology of National Health Commission, Beijing, 100730, China.; 2 Chinese Academy of Medical Sciences & Peking Union Medical College, Beijing, 100730, China.; 3 Department of Cardiology, Beijing Hospital, National Center of Gerontology, Institute of Geriatric Medicine, Chinese Academy of Medical Sciences & Peking Union Medical College, Beijing 100730, China.; 4 Biomedical Pioneering lnnovation Center, School of Life Sciences, Peking University, Beijing 100871, China.; 5 Peking University Fifth School of Clinical Medicine, Beijing, 100730, China.

**Keywords:** Madecassic acid, Doxorubicin, Cardiotoxicity, SIRT1, Chemotherapy

## Abstract

Doxorubicin (DOX) is an anthracycline that has excellent anticancer effects during tumor chemotherapy, but it can cause cardiotoxic effects and its clinical use has been limited. Therefore, finding new drugs or methods to prevent or reverse the cardiac damage caused by DOX therapy in cancer patients is essential. Previous studies have identified potential cardioprotective effects of *Centella asiatica* (*C. asiatica*), and madecassic acid (MA) is a pentacyclic triterpenoid derived from *C. asiatica*. However, the pharmacological effects of MA on the heart and tumors during tumor chemotherapy are not fully understood. The aim of this study was to investigate the pharmacological function and molecular mechanisms of MA in the heart and tumor during chemotherapy.

In a DOX-induced acute heart failure mouse model and a cardiomyocyte injury model, MA reduced cardiomyocyte oxidative stress and the inflammatory response, improved mitochondrial function, and attenuated autophagic flux blockade and apoptosis. Interestingly, MA significantly increased the expression and activity of SIRT1. When SIRT1 was knocked down, the protective effect of MA on cardiomyocytes was significantly inhibited, suggesting that MA may exert cardioprotective effects through the SIRT1 pathway. Interestingly, in contrast to its cardioprotective effect, MA could synergize with DOX and significantly contribute to the anticancer chemotherapeutic effect of DOX by inhibiting proliferation, migration and invasion; promoting apoptosis; and suppressing tumor progression by inhibiting the expression of the DDX5 pathway in tumor cells. Here, we identified the pharmacological functions of the pentacyclic triterpenoid MA in ameliorating DOX-induced cardiotoxicity and enhancing the antitumor efficacy of DOX.

## 1. Background

In recent years, the cancer incidence and mortality have increased due to an aging population and environmental pollution. Doxorubicin (adriamycin, DOX) is one of the earliest and most effective chemotherapeutic agents and is recognized for its potent antitumor activity. As an anthracycline antibiotic derived from Streptomyces species^1^, DOX is primarily used to treat acute leukemia, malignant lymphoma, squamous cell carcinoma, and hepatocellular carcinoma, among other conditions^2^-^4^. However, its clinical use is significantly limited by side effects such as hematopoietic suppression, cardiotoxicity, and hepatotoxicity^5^, ^6^. Among these side effects, DOX-induced cardiotoxicity is particularly severe, potentially leading to heart failure and even death^5^, ^7^. When cumulative doses of anthracyclines exceed 400 mg/m^2^, they have the potential to cause cardiotoxic effects and may also cause irreversible cardiomyopathy and heart failure, which is a major factor limiting the use of DOX in chemotherapy^8^. Therefore, the search for drugs or treatments that can mitigate DOX-induced cardiotoxicity has important clinical preventive and therapeutic implications for the chemotherapy of cancer patients.

Approximately one-third of all clinical drugs are derived from natural products, highlighting significant interest in medicinal plants^9^. Among these, terpenoids have garnered considerable attention because of their pharmacological activities^10^. Pentacyclic triterpenoids, in particular, are known for their potent anti-inflammatory, anticancer, and cardiovascular protective properties^11^. MA is a natural triterpenoid compound extracted from the traditional medicinal plant *C. asiatica*, that is widely distributed worldwide^12^. Recent studies have shown that MA possesses various pharmacological activities, including wound healing, anticancer, antioxidant, anti-inflammatory, and antidiabetic effects^12^-^14^. Although some studies have reported its effects, its pharmacological functions and signaling pathways are poorly understood and require further research.

Moreover, many natural compounds have been identified as agonists of SIRT1, exerting cardioprotective effects by increasing the expression and/or activity of SIRT1^15^. SIRT1 is an NAD^+^-dependent deacetylase involved in the regulation of a variety of pathological and physiological processes, including cell proliferation, differentiation, autophagy, and oxidative stress^16^. Increasing evidence indicates that SIRT1 plays a protective role in pathological processes such as myocardial ischemia/reperfusion injury, cardiac hypertrophy, diabetic cardiomyopathy and cardiac aging^17^-^19^. Therefore, changes in SIRT1 levels have significant effects on the cardiomyocyte status and cardiac function^20^. SIRT1 has emerged as an important target for the inhibition of DOX-induced cardiotoxicity^21^, ^22^. Some studies have shown that the expression and activity of SIRT1 are downregulated during DOX-induced cardiotoxicity and that the overexpression of SIRT1 can exert cardioprotective effects through multiple signaling pathways to counteract DOX-induced cardiotoxicity^16^, ^21^, ^22^.

DDX5 is a member of the DEAD-box protein subfamily. The protein sequences and cellular functions of the members of this subfamily are highly conserved^23^, ^24^. DDX5 is involved in almost all RNA metabolic processes, such as alternative splicing of mRNAs, miRNA maturation, ribosome biogenesis, degradation of mRNAs, and interactions with lncRNAs, and functions as a transcriptional cofactor involved in regulating the activity of transcription factors involved in transcription^23^-^25^. DDX5 is highly expressed in colon, prostate, breast, brain, lung, and gastric cancers, as well as leukemia, and inhibition of DDX5 expression slows tumor cell growth, suggesting that DDX5 is required for cancer development^23^, ^26^.

Here, we found that MA, a natural triterpenoid plant compound, exerts cardioprotective effects by promoting SIRT1 expression and activity and reducing DOX-induced autophagy pathway blockade and apoptosis in cardiomyocytes. Additionally, MA inhibits cancer cell proliferation and enhances the chemotherapeutic effect of DOX. Therefore, MA has the dual effects of anticancer potentiation and cardioprotection. It may have good potential in the future clinical prevention and treatment of cardiovascular disease and in the synergistic application with chemotherapy for the treatment of malignant tumors.

## 2. Material and methods

### 2.1 Animal experiments

C57BL/6J male mice, 8‒10 weeks old, were provided by SPF (Beijing) Biotechnology Co. Ltd and housed in a specific pathogen-free platform with controlled temperature (22‒24°C), humidity (55%‒60%), and a 12-hour light-dark cycle, with free access to food and water. Animal experiments were conducted in accordance with the Guide for the Care and Use of Laboratory Animals (NIH Publication, Revised 2011) and approved by the Animal Use and Care Committee of Peking University (LA2021576). Mice were randomly divided into four groups: ① control group (CON), ② MA group, ③ DOX group, and ④ DOX+MA group. DOX was purchased from Cell Signaling Technology (USA) and dissolved in saline. Cardiotoxicity was induced in the DOX group by intraperitoneal injection of DOX at a single dose of 2.5 mg/kg every other day for a total of six injections. Animals in the MA and DOX+MA groups received 25 mg/kg of MA (Solarbio, China) daily by gavage, with the MA gavage administered two days prior to the start of the intraperitoneal DOX injections. The control group received an equal volume of saline injection or blank vehicle gavage. Mice were anesthetized with 3‒4% isoflurane in oxygen and euthanized by cervical dislocation 12 days after the first DOX injection. Peripheral blood was collected for analysis. Hearts were perfused with saline, weighed, and placed in 4% paraformaldehyde or liquid nitrogen for further analysis.

### 2.2 Echocardiography

At the end of the experiment, mice were anesthetized with 1%‒1.5% isoflurane and body temperature was maintained with a heating pad. Cardiac function was assessed when the heart rate stabilized at 400‒500 beats/min. Echocardiography operators were blinded to animal group, and measurements were performed using a Vevo 3100 imaging system (Visual Sonics), as previously reported^27^.

### 2.3 Measurement of Lactate Dehydrogenase (LDH) activity, creatine kinase (CK) level and Malondialdehyde (MDA) level

After mice were anesthetized, whole blood was collected from the right ventricle and allowed to stand at room temperature for 1 hour, then centrifuged at 4°C for 15 minutes at 1000g to obtain serum. LDH and CK levels were determined using a LDH assay kit (Cat. No. S03034) and a CK assay kit (Cat. No. S03024) (Rayto Life and Analytical Sciences Ltd.). All ELISA experiments were performed according to the manufacturer's protocols^20^. The extraction and detection of MDA in heart tissues were performed according to the manufacturer's protocol of the MDA assay kit (Cat. No. A003-1) from Nan Jing Jian Cheng Bioengineering Institute, and the measurement of MDA levels in H9c2 cells were performed according to MDA assay kit (Cat. No. BC0020) from Beijing Solarbio Co^28^.

### 2.4 Cell culture, isolation of Neonatal mouse ventricular myocytes (NMVMs), and viability assays

H9c2, HepG2, A431, and Huh-7 cell lines were purchased from the American Type Culture Collection (ATCC). Cell lines were cultured in Dulbecco's modified Eagle's medium (Gibco, USA) containing 10% fetal bovine serum (Gibco, USA) and antibiotics (100 U/mL penicillin G and 0.1 mg/mL streptomycin) at 37°C in an incubator containing 5% CO_2_ and 95% air (v/v). Cells were seeded in 6-well plates containing 10% FBS (Gibco, USA) in DMEM for 24 h, then synchronized with DMEM containing 0.5% FBS for 24 h, and then treated with DOX for 24 h. In the treatment of tumour cell lines, MA was dosed simultaneously with DOX without pretreatment.

NMVMs were isolated from 1-3 day old C57BL/6J mice. Cardiomyocytes were plated in culture dishes at a density of 6.6×10^4^ cells/cm^2^ using DMEM and M199 medium (Sigma-Aldrich) at a volume ratio of 3:1 and supplemented with 5% FBS and 10% equine serum (Sigma-Aldrich) in the presence of 0.1 mM 5-bromo-2-deoxyuridine (Sigma-Aldrich) and antibiotics (100 U/mL penicillin G and 0.1 mg/mL streptomycin). All cells were cultured at 37°C in an incubator containing 5% CO_2_ and 95% air (v/v)^27^.

Cell-Counting-Kit-8 (CCK8, Solarbio, China) was used to measure the number of live cells as previously described. Briefly, 10 μL of CCK8 solution was added to a 96-well plate seeded with cells and incubated for 4 hours. The absorbance was recorded at 450 nm.

### 2.5 *In situ* detection of reactive oxygen species (ROS)

After the experiment, H9c2 cells or cardiomyocytes were incubated with 10 μmol/L dihydroethidium (DHE, Sigma-Aldrich) in a dark humidified chamber in a 37°C incubator for 30 minutes, and ROS production was observed by the red fluorescence method under a fluorescence microscope. Fluorescence intensity was analyzed using ImageJ software as previously reported^20^.

### 2.6 Sirius red staining, Hematoxylin-eosin (H&E) staining, Masson staining

Hearts were sectioned as described in our previous study. Briefly, hearts were excised, fixed in 4% paraformaldehyde, and embedded in paraffin. Mouse hearts were sectioned at 5 μm thickness. Cardiomyocyte morphology and inflammatory cell infiltration were observed by H&E staining. Collagen deposition and myocardial fibrosis were detected by Masson's and Sirius red staining. Images were analyzed using Image J software to quantify fibrotic areas^20^.

### 2.7 TUNEL staining

TUNEL staining (Roche) was performed according to previously reported methods. Cell culture slides fixed in 4% paraformaldehyde for 30 minutes at room temperature and permeabilized with 0.1% TritonX-100 were subjected to TUNEL staining according to the instructions of the Roche Cell Death Assay Kit. Cell nuclei were stained with 10mM Hoechst 33342 for 1 minute and then observed by inverted fluorescence microscopy. The percentage of TUNEL-positive cells was calculated by randomly selecting 10 cell areas from each culture dish; each data point represents the results of at least 2000-2500 cells in 3 independent experiments^20^.

### 2.8 Mitochondrial staining and analysis of mitochondrial fission

Mitochondrial staining was modified as previously described. Briefly, H9c2 cells were seeded on Crawler dishes. After treatment, they were stained with 0.02 µM Mito-Tracker Red CMXRos (Molecular Probes) for 20 min. Mitochondria were imaged by laser scanning confocal microscopy^27^.

### 2.9 Measurement of SIRT1 activity

The treated cells were assayed using a colourimetric quantitative assay kit for SIRT1 activity (Shanghai Haling, Cat. HL50287.1 v.A). The kit is an assay designed to detect SIRT1 activity by means of a colourimetric probe.

### 2.10 Assessment of Mitochondrial Membrane Potential by JC-1 in NMVMs

When the mitochondrial membrane potential is high, JC-1 aggregates in the mitochondrial matrix, forms polymers, and stains red. Conversely, JC-1 does not aggregate, forms monomers, and shows green fluorescence. After the cells were treated and replaced with fresh medium, JC-1 was added at a concentration of 2µg/ml and co-incubated for 20 minutes at 37℃. The ratio of red fluorescence to green fluorescence was observed using an inverted fluorescence microscope and analyzed and calculated using Image J^27^. Mitochondrial Membrane Potential Assay Kit with JC-1 purchased from Beijing Solarbio Co.

### 2.11 Monodansyl cadaverine (MDC) staining

MDC staining was performed according to the manufacturer's protocol. Briefly, H9c2 cells were processed and then incubated in 0.05 mM MDC in DMEM at 37°C for 30 min protected from light, then washed three times with PBS at room temperature and replaced with fresh DMEM medium. Finally, autophagic vacuoles were observed under an inverted microscope, using excitation and emission wavelengths of MDC of 335 nm and 512 nm, respectively^29^.

### 2.12 GFP-LC3 adenovirus and GFP and mRFP tandemly tagged LC3 adenovirus transfection for autophagy assay and autophagic flux assay

NMVMs were cultured in a 6-well plate for 24 hours and then transfected with mRFP-GFP-LC3-Adv for 24 hours at a multiplicity of infection (M.O.I.) of 10. After transfection, the cells were treated with MA or DOX. The cells were then observed by inverted fluorescence microscopy. The number of RFP and GFP spots in the 5 fields of view was counted, and at least 50 cells were counted in each group, as previously reported^30^.

### 2.13 Transfection of siRNA

Rat siRNA negative control (NC, sense: 5'-UUCUCCGAACGUGUCACGUTT-3', antisense: 5'-ACGUGACACGUUCGGAGAATT-3') and rat SIRT1 siRNA (si-SIRT1, sense: 5'-CCAGUAGCACUAAUUCCAATT-3', antisense: 5'-UUGGAAUUAGUGCCACUGGTT-3') were chemically synthesized by Genepharma. Mouse siRNA Negative control (Nci, Cat. No. sc-37007, Santa Cruz, USA) and mouse SIRT1 siRNA (Cat. No. sc-40987, Santa Cruz, USA) were purchased from Santa Cruz Co. When the cell density was cultured to 70%, Nci and si-SIRT1 were transfected into cells using HiPerFect transfection reagent (QIAGEN). After 48 hours of transfection, the transfected cells were replaced with fresh DMEM medium. The cells were then pretreated with MA for 12 hours, followed by the addition of DOX for 24 hours^20^.

### 2.14 DNA Fragmentation analysis (DNA laddering)

1 × 10^7^ cells were harvested and digested overnight at 37°C with cell lysis buffer containing 10% SDS and protease K. DNA was extracted with phenol/CHCl3/isoamyl alcohol (25:24:1). After addition of sodium acetate and ethanol, the extracted DNA was stored at -20°C for one week. The DNA was then washed once with 70% ethanol and dissolved in TE buffer. DNA fragments were detected by agarose gel electrophoresis and stained with ethidium bromide as previously reported^20^.

### 2.15 Quantitative real-time PCR

Total RNA was extracted from cultured cardiomyocytes or myocardial tissue using TRIzol reagent (Invitrogen, Carlsbad, CA, USA). cDNA was synthesised from equal amounts of total RNA per sample using the cDNA First Strand Synthesis Kit (New England Biolabs). Quantitative real-time PCR was performed using the Applied Biosystems Quant Studio 3 Real-Time PCR System (Thermo Fisher Scientific, Waltham, MA, USA) and a reaction mixture containing SYBR Green (TaKaRa, Japan). The real-time PCR profile was as follows: 95°C for 120 s, 40 cycles at 95°C for 5 s, 60°C for 34 s. The amount of SYBR Green was measured at the end of each cycle. Gene expression was normalized to the level of *Gapdh*. A total of 3 replicates were performed for each reaction and analysis was performed by the 2^(-ΔΔCt) method. Primer sequences for the target genes included: *Gapdh*, 5'-AACTTTGGCATTGTGGAAGG-3' and 5'-ACACATTGGGGGTAGGAACA-3'; *Nppa*, 5'-GATAGATGAAGGCAGGAAGCCGC-3' and 5'-AGGATTGGAGCCCAGAGTGGACTAGG-3'; *Myh7*, 5'-TTGGGAAATTCATCCGAATC-3' and 5'-CCAGAAGGTAGGTCTCTATG-3'; *Il1b*, 5'-CAGGCAGGCAGTATCACTCA-3' and 5'-TGTCCTCATCCTGGAAGGTC-3'; *Il6*, 5'-CCGGAGAGGAGACTTCACAG-3' and 5'-TCCACGATTTCCCAGAGAAC-3'; *Tgfb1*, 5'-TGATACGCCTGAGTGGCTGTCT-3' and 5'-CACAAGAGCAGTGAGCGCTGAA-3'.

### 2.16 Western blotting assay

Cells were lysed with cell lysis buffer containing complete protease inhibitor, phosphatase inhibitor and PMSF. The BCA method was used to determine the protein concentration of each group. 30 μg of protein samples were loaded on SDS-PAGE for electrophoresis and then transferred to PVDF membrane. The membranes were incubated with 5% skim milk for 1 hour at room temperature, then incubated with primary antibody overnight at 4°C and rinsed 6 times with TBST, then incubated with secondary antibody for 2 hours at room temperature and rinsed 6 times with TBST. Detection was performed using enhanced chemiluminescence reagents, and photographs were taken using a Vilber Bio Imaging system. GAPDH was used as a protein loading control. Finally, gray-scale analysis of the images was performed using ImageJ software (http://rsb.info. nih.gov/ij/), as previously reported^20^.

### 2.17 Statistical analysis

Data analysis was performed with GraphPad Prism 8.0. All data are presented as mean ± SEM. Differences between two groups were analyzed by Student's t-test, and differences between multiple groups were analyzed by one-way ANOVA with Bonferroni correction. Differences between groups were considered significant at *P* < 0.05.

## 3. Results

### 3.1 MA attenuates DOX-induced myocardial injury in cardiomyocytes

**Figure 1:**
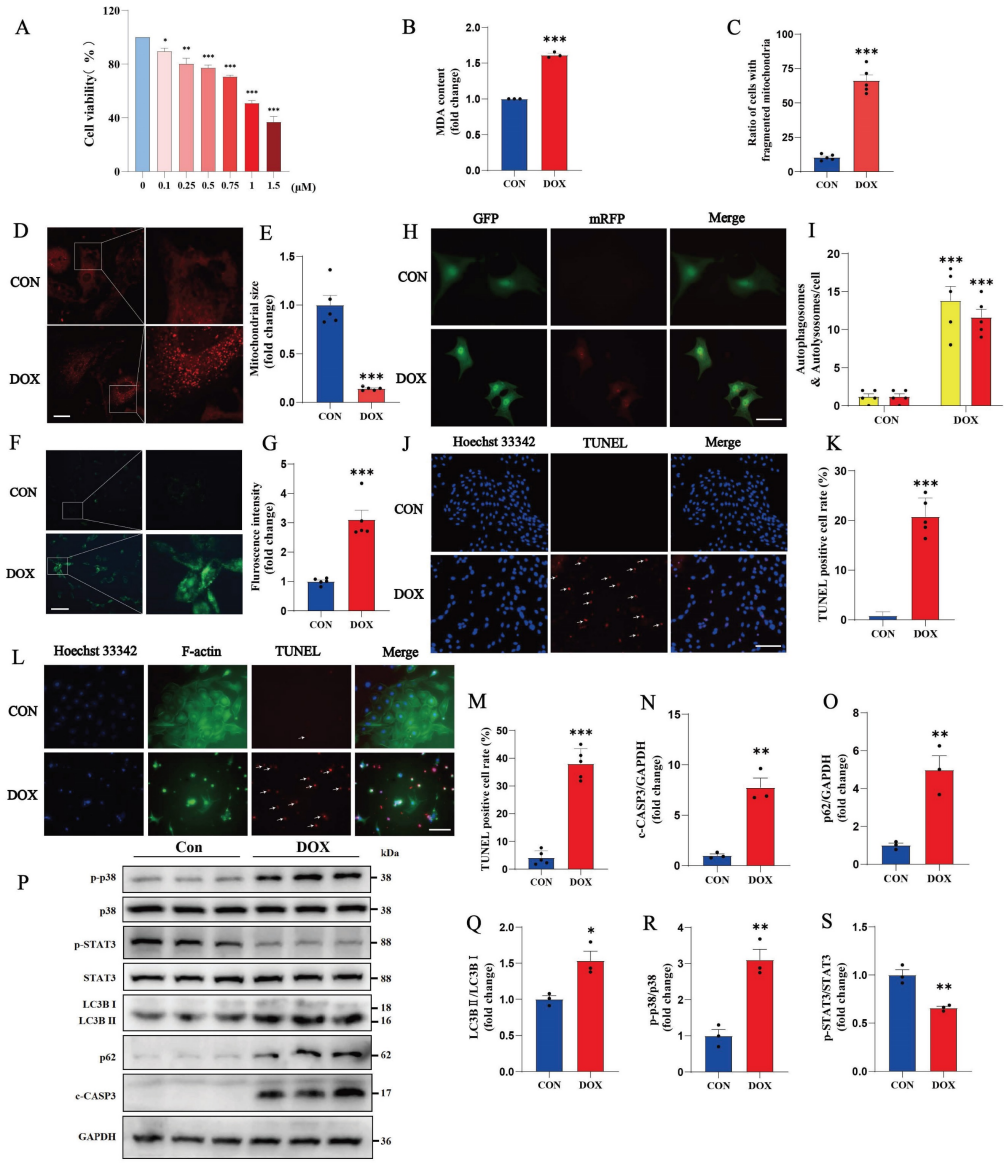
DOX causes oxidative stress, mitochondrial damage, blockade of autophagic flux and apoptosis in cardiomyocytes. (A) Cell viability was determined by a CCK-8 assay in the control and DOX groups. (B) Measurement of MDA levels in the Con and DOX groups (n = 3). (C-E) Images of the morphology of MitoTracker Red-stained mitochondria (n=5). Scale bar: 20 µm. (F, G) NMVMs were transfected with 10 M.O.I. of tandem fluorescence-labeled LC3 adenovirus (mRFP-GFP-LC3) to detect autophagic flux. The red color represents the number of red fluorescence spots per cell, which represents the number of intracellular autolysosomes. Yellow represents the number of spots of yellow fluorescence per cell, indicating the number of intracellular autophagosomes (n=5). Scale bar: 50 µm. (H, I) Autophagy levels in H9c2 cells were detected via the MDC method (n=5). Scale bar: 100 µm. (J, K) Representative images of TUNEL and Hoechst 33342 staining of H9c2 cells (n=5). Scale bar: 100 µm. (L, M) Representative image of NMVMs stained with Hoechst 33342, F-actin and TUNEL (n=5). Scale bar: 100 µm. (N-S) Phosphorylated and total p38, total STAT3, LC3B I, LC3B II, p62 and c-CASP3 expression levels were detected by Western blotting (n≥3). ns: not significant, *P < 0.05, **P < 0.01, and ***P < 0.001 compared with the CON group.

Cardiac cytotoxicity models of H9c2 cells or NMVMs were established with DOX treatment. After 0.75 μM DOX treatment, the viability of H9c2 cells was significantly reduced, and the cell morphology was disorganized and altered. A CCK-8 cell viability assay revealed a cell death rate of 29.38% after treatment with 0.75 μM DOX (Fig. 1 A). Compared with the control, DOX induced an increase in MDA production in H9c2 cells compared to the control (Fig. 1B). Mitochondrial staining revealed increased mitochondrial fission, a decreased mitochondrial size and an uneven distribution in NMVM cells after DOX treatment (Fig. 1C-E). An increase in the number of autophagic vesicles in DOX-treated cardiomyocytes was detected via the MDC assay (Figure 1F, G). After the cardiomyocytes were transfected with mRFP-GFP-LC3-Adv, a blockage of autophagic flux was observed in the NMVMs from the DOX group (Figure 1H, I). TUNEL staining revealed nuclear chromatin condensation and increased numbers of apoptotic bodies in H9c2 cells (Fig. 1J, K). We obtained similar results using NMVMs treated with 0.3 µM DOX (Fig. 1L, M).

Next, in DOX-induced cardiomyocytes, an increase in the ratio of LC3B II to LC3B I was detected, suggesting that the level of autophagy in cardiomyocytes was significantly increased. As an autophagy-specific substrate, p62 can interact with LC3B II and participate in the degradation of autophagosomes. When the autophagosome fuses with the lysosome to degrade the contents of the autophagosome, the cellular autophagic flux increases, and the level of p62 decreases due to its increased consumption; on the other hand, when the fusion of the autophagosome and lysosome is impaired, p62 cannot participate in the autophagic lysosomal degradation process, and thus the expression of p62 increases. In DOX-treated cardiomyocytes, both LC3B II/I and p62 levels were significantly increased, indicating that the fusion process of autophagosomes and lysosomes was impeded and that autophagic flux was blocked (Fig. 1O-Q).

In addition, the proinflammatory p38 signaling pathway was significantly inactivated, and the anti-inflammatory and antioxidant STAT3 signaling pathways were inhibited in the DOX group (Fig. 1 P, R, S). This evidence suggests that DOX successfully induces inflammation, oxidative stress, mitochondrial damage, autophagic flux blockade and apoptosis in cardiomyocytes.

**Figure 2:**
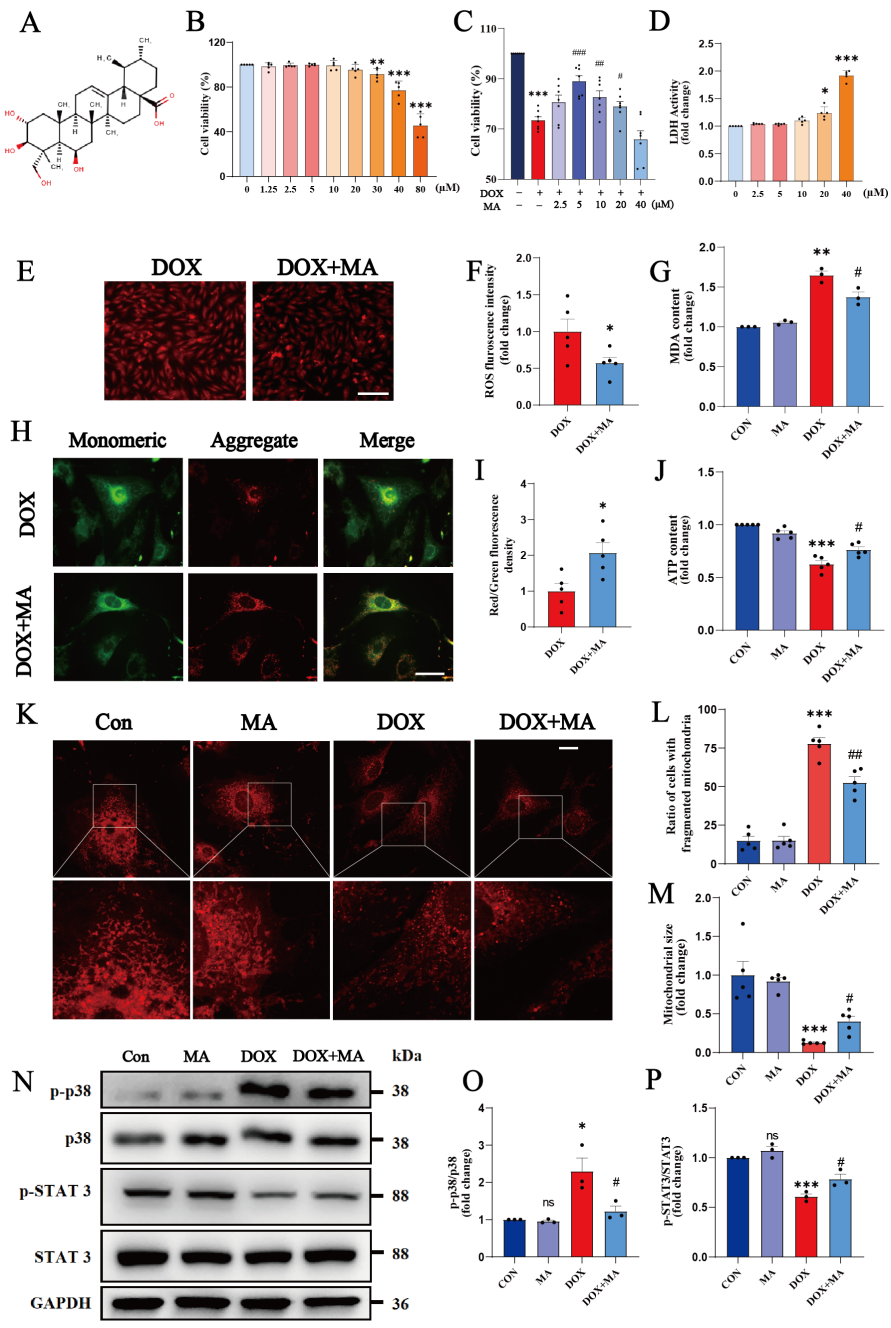
MA attenuates DOX-induced oxidative stress and mitochondrial damage in cardiomyocytes. (A) Chemical structure of MA. (B) Cell viability was determined using a CCK-8 assay after H9c2 cells were treated with 0-80 μM MA for 24 hours (n=5). (C) H9c2 cells were pretreated with 0-40 μM MA for 12 hours and then treated with DOX. Cell viability was measured with a CCK-8 assay (n=7). (D) LDH release assay (n=5). (E, F) DHE staining of H9c2 cells in the DOX and DOX+MA groups (n=5). Scale bar: 100 µm. (G) Measurement of MDA levels in the Con, MA, DOX, and DOX+MA groups (n=3). (H, I) Detection of the mitochondrial membrane potential by JC-1 staining in NMVMs from the DOX and DOX+MA groups (n=5). Scale bar: 50 µm. (J) ATP levels measured in the Con, MA, DOX, and DOX+MA groups (n=5). (K-M) Images showing the morphology of MitoTracker Red-stained mitochondria (n=5). Scale bar: 20 µm. (N-P) Phosphorylated and total p38 and STAT3 expression levels were detected by Western blotting (n≥3). ns: not significant, *P < 0.05, **P < 0.01, and ***P < 0.001 compared with the CON group. ^#^*P* < 0.05, ^##^*P* < 0.01, and ^###^*P* < 0.001 compared with the DOX group.

We screened 34 natural compounds with antioxidant and/or anti-inflammatory properties, most of which, such as delphinidin, apigenin, and Tremella polysaccharide, had no significant protective effect on DOX-induced myocardial toxicity. We also screened some of the natural compounds with protective effects, such as dihydromyricetin, MA, and Astragalus polysaccharide. Finally, we focused on the most potent pentacyclic triterpenoid among the natural terpene compounds, MA, a triterpenoid found in *C. asiatica* with a wide range of biological activities (Fig. 2A).

First, we investigated the cytotoxicity of various concentrations of MA to cardiomyocytes. Cell viability assays revealed no statistically significant difference in cell viability after the treatment of H9c2 cells with 0-20 μM MA for 24 h, indicating that 0-20 μM MA was not significantly cytotoxic (Fig. 2B). We pretreated H9c2 cells with different concentrations of MA for 12 hours prior to DOX treatment to investigate whether MA had a protective effect on DOX-induced cardiomyocyte injury. A cell viability assay revealed that 5 μM MA significantly attenuated DOX-induced cardiomyocyte injury and cell death (Fig. 2C). LDH release assay revealed that MA is not significantly damaging to cardiomyocytes at less than 20µM (Fig. 2D). Therefore, 5 μM MA was used in a subsequent study. Pretreatment of H9c2 cells with MA effectively inhibited DOX-induced ROS accumulation and MDA production (Fig. 2E-G). JC-1 staining and MitoTracker staining revealed that MA treatment attenuated the DOX-induced decrease in the mitochondrial membrane potential, reduced mitochondrial fission, and maintained the number and morphology of the mitochondria (Fig. 2H-M). In addition, the proinflammatory p38 pathway was inhibited, whereas the anti-inflammatory and antioxidant STAT3 pathways were significantly activated by MA pretreatment (Fig. 2N-P). These results indicate that pretreatment with MA can significantly inhibit DOX-induced oxidative stress, mitochondrial damage, and inflammatory responses.

**Figure 3:**
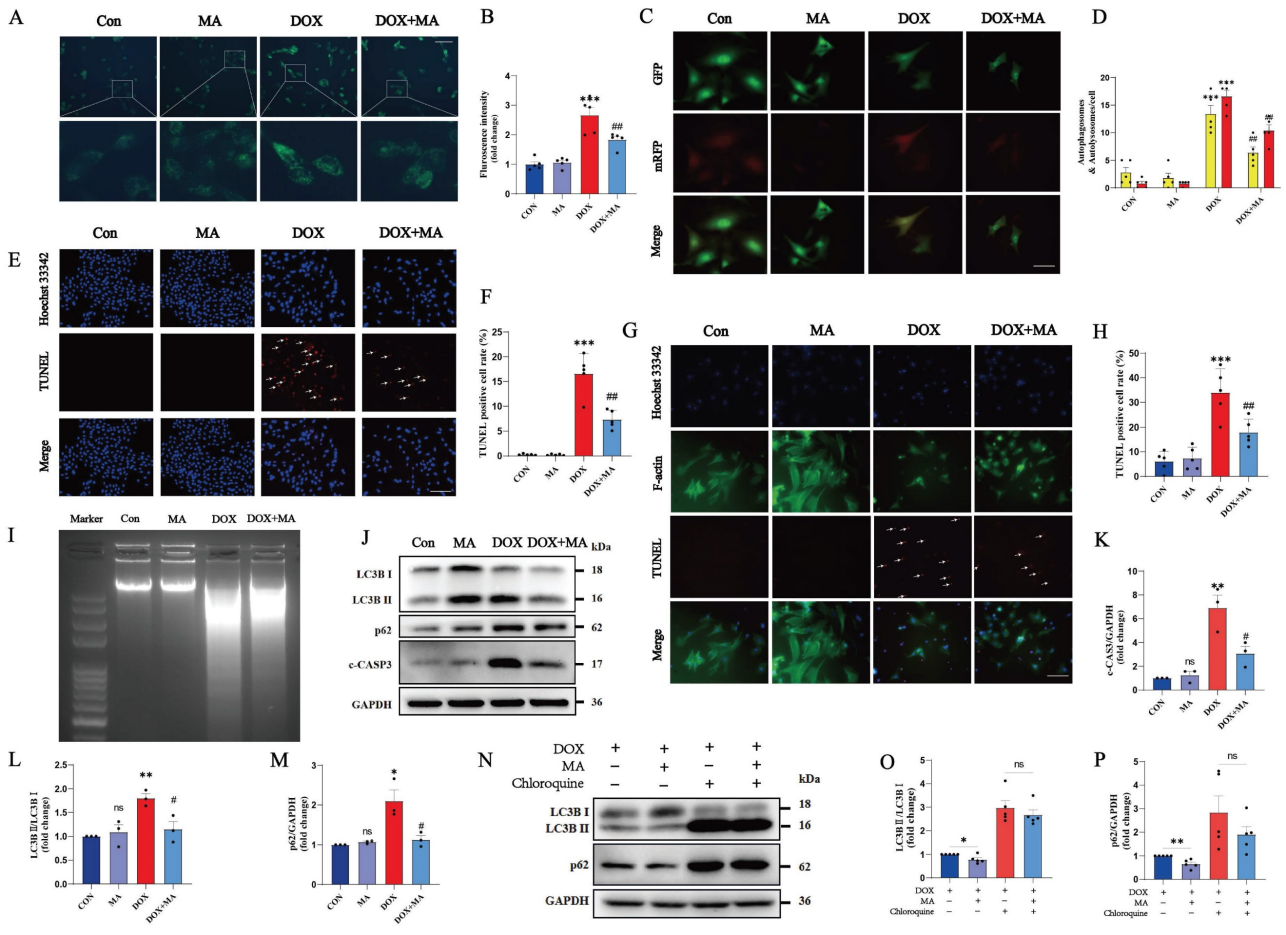
MA inhibits DOX-induced autophagic flux blockade and apoptosis in cardiomyocytes. (A, B) Autophagy levels in H9c2 cells were detected via an MDC assay (n=5). Scale bar: 100 µm. (C, D) NMVMs were transfected with 10 M.O.I. of tandem fluorescence-labeled LC3 adenovirus (mRFP-GFP-LC3) to detect autophagic flux. The red color represents the number of red fluorescence spots per cell, which represents the number of intracellular autolysosomes. Yellow represents the number of spots of yellow fluorescence per cell, indicating the number of intracellular autophagosomes (n=5). Scale bar: 50 µm. (E, F) TUNEL and Hoechst 33342 staining (n=5). Scale bar: 100 µm. (G, H) Representative images of Hoechst 33342, F-actin and TUNEL staining of NMVMs (n=5). Scale bar: 100 µm. (I) Images of DNA laddering in the Con, MA, DOX, and DOX+MA groups. (J‒M) The expression levels of LC3B I, LC3B II, p62 and c-CASP3 were detected by Western blotting (n=3). (N‒P) The expression levels of LC3B I, LC3B II and p62 were detected by Western blotting (n=5). GAPDH was used as a loading control. ns: not significant, *P < 0.05, **P < 0.01, and ***P < 0.001 compared with the control group. ^#^*P* < 0.05, ^##^*P* < 0.01, and ^###^*P* < 0.001 compared with the DOX group.

In the DOX group, the number of autophagic vesicles increased significantly, which was mainly due to the abnormal autophagic flux blockade. Pretreatment with MA attenuated the DOX-induced blockade of autophagic flux (Fig. 3A-D). Moreover, TUNEL and DNA laddering experiments also revealed that MA pretreatment effectively reduced DOX-induced apoptosis in both H9c2 cells (Fig. 3E, F, I) and NMVMs (Fig. 3G, H). In addition, MA pretreatment significantly decreased the expression of c-CASP3, confirming the antiapoptotic effect of MA on cardiomyocytes (Fig. 3J, K). Western blot analysis of LC3B II/I and p62 revealed that MA pretreatment attenuated DOX-induced autophagic flux blockade (Fig. 3J, L, M). In contrast, the autophagy inhibitor chloroquine completely blocked the protective effect of MA on autophagy (Fig. 3N-P). These data suggest that MA pretreatment exerts cardioprotective effects by alleviating DOX-induced autophagic flux blockade and reducing cell apoptosis.

### 3.2 MA is a small-molecule SIRT1 agonist derived from natural products

**Figure 4:**
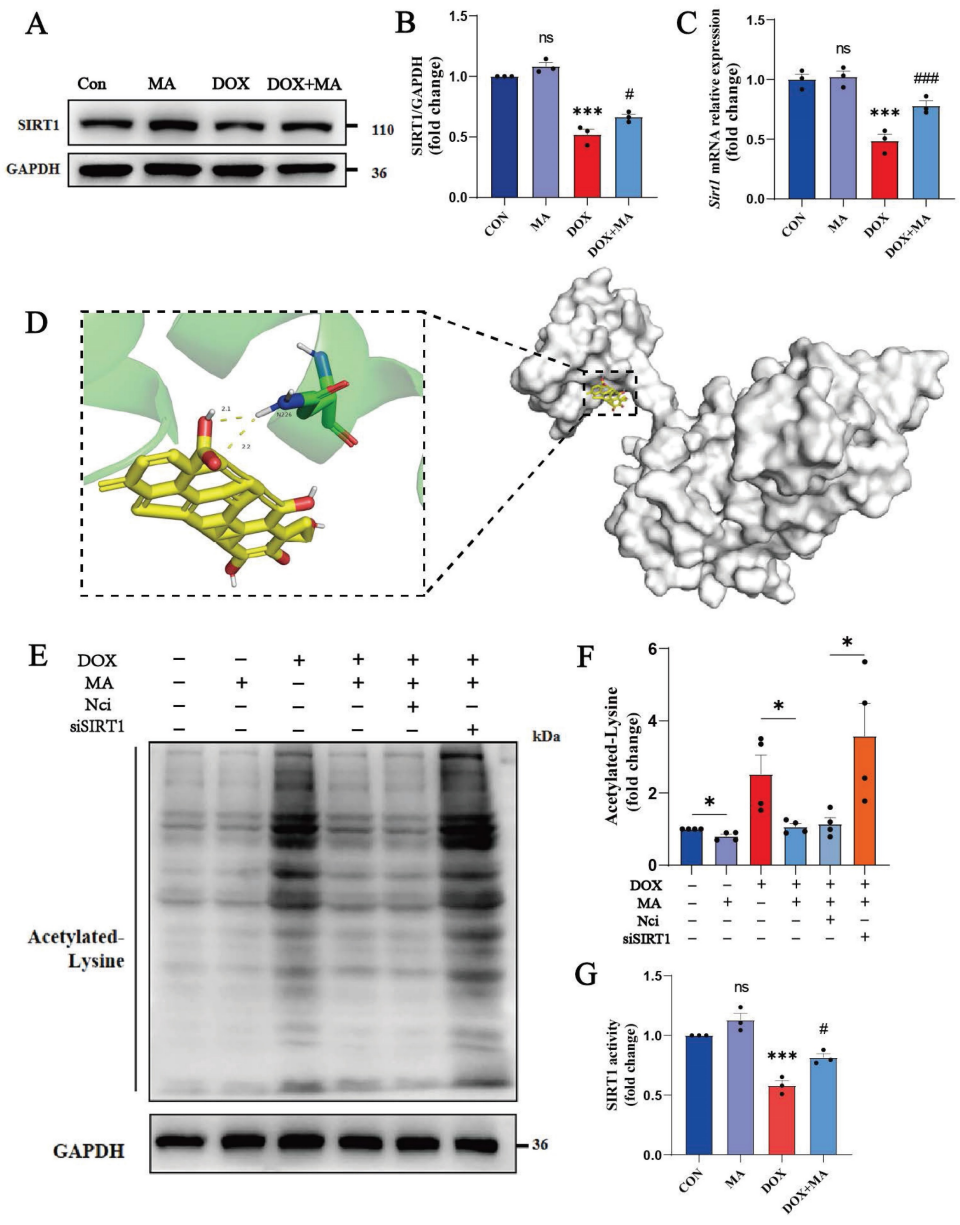
MA is a small-molecule agonist of SIRT1. (A, B) SIRT1 protein expression in H9c2 cells was detected by Western blotting. GAPDH was used as a loading control (n=3). (C) Real-time PCR was performed to detect* Sirt1* expression in H9c2 cells (n=3). (D) Molecular docking model of MA and SIRT1. The large molecule in the right panel is the SIRT1 protein, and the small yellow molecule in the left panel is MA, which forms a hydrogen bond with amino acid residue ASN-226 at the end of SIRT1. (E, F) The expression of acetylated lysine protein was detected by Western blotting (n=3). (G) The activity of SIRT1 in NMVMs (n=3). ns: not significant, *P < 0.05, **P < 0.01, ***P < 0.001. ^#^*P* < 0.05, ^##^*P* < 0.01, and ^###^*P* < 0.001.

We screened key signaling molecules involved in cardiomyocyte oxidative stress, cell survival and cell death to explore the pharmacological mechanisms by which MA protects the myocardium from DOX damage. Among these factors, the key cardiomyocyte protective factor SIRT1 showed the most significant changes. Our study revealed that SIRT1 expression was significantly reduced in DOX-treated cardiomyocytes, whereas MA pretreatment significantly upregulated SIRT1 expression (Fig. 4A, B, C).

We performed molecular docking studies to analyze whether MA matches the spatial structure, electrical properties, and hydrophobicity of SIRT1. Software such as AutoDock 4 and PyMOL 2.6 was used for molecular docking and visualization. The docking sites of MA and SIRT1 were simulated using a semiflexible molecular docking method. The PDB ID of SIRT1 is 4ZZH. Previously, Han *et al.* reported that small molecule SIRT1 activators bind to the N-terminus of SIRT1, which in turn activates the catalytic activity of SIRT1. Therefore, we adjusted the docking cassette to wrap only the N-terminal region of SIRT1. We found that MA could directly bind to SIRT1, and the docking binding energy was 7.42 kJ/mol, indicating that the two molecules had good binding. MA binds to the hydrophobic pocket at the N-terminal end of SIRT1. MA forms a hydrophobic bond with the hydrophobic site of SIRT1, and MA forms a hydrogen bond with the hydrophilic residue of ASN-226 of SIRT1, which plays a key role in SIRT1 activation (Fig. 4D). In addition, the activity of SIRT1 was detected through tyrosine acetylation, and MA was found to perform its biological functions through the activation of SIRT1 (Fig. 4E, F, G). Therefore, MA has the potential to be a new small-molecule SIRT1 agonist.

### 3.3 SIRT1 knockdown reverses the protective effects of MA on DOX-induced cytotoxicity

**Figure 5:**
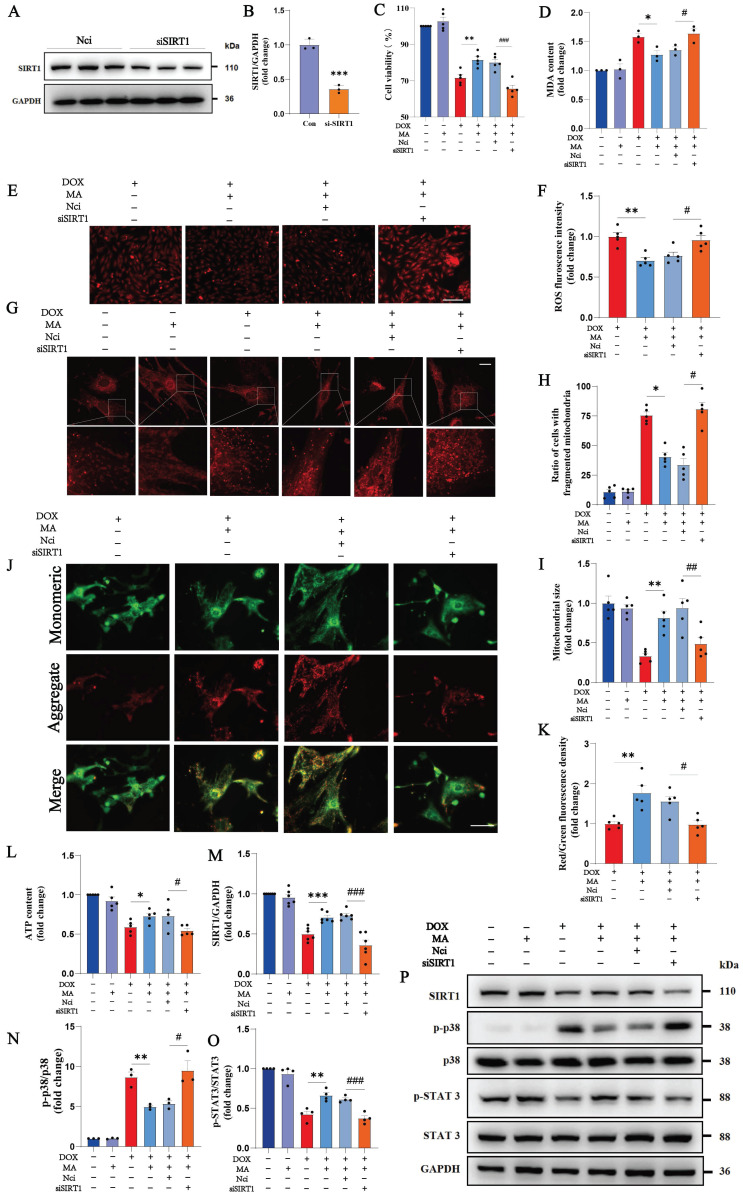
Knockdown of SIRT1 reverses the cardiocytoprotective effect of MA. (A, B) Detection of SIRT1 protein expression in NCi- and si-SIRT1-transfected H9c2 cells after 48 h. GAPDH was used as a loading control (n=3). (C) The viability of H9c2 cells was detected via a CCK-8 assay (n=5). (D) Measurement of the MDA content in each group (n=3). (E, F) ROS production was analyzed by DHE staining (n=5). Scale bar: 100 µm. (G‒I) Images showing the morphology of MitoTracker Red-stained mitochondria (n=5). Scale bar: 20 µm. (J, K) The mitochondrial membrane potential in NMVMs from each group was detected using JC-1 staining (n=5). Scale bar: 50 µm. (L) ATP levels in each group (n=5). (M‒P) The expression levels of SIRT1, phosphorylated and total p38, and phosphorylated and total STAT3 were detected by Western blotting (n=3‒6). GAPDH was used as a loading control. ns: not significant, *P < 0.05, **P < 0.01, ***P < 0.001. ^#^*P* < 0.05, ^##^*P* < 0.01, and ^###^*P* < 0.001.

We knocked down SIRT1 in cardiomyocytes using a SIRT1 siRNA to further investigate the role of SIRT1 in the protective effects of MA on cardiomyocytes. MA pretreatment was administered 12 hours before DOX administration. As shown in Fig. 5A-B, the knockdown efficiency of the SIRT1 siRNA was approximately 60%. When SIRT1 was knocked down, cell viability assays revealed that the protective effect of MA on DOX-treated cardiomyocytes was completely inhibited (Fig. 5C). ROS and MDA production were also significantly increased in H9c2 cells after SIRT1 knockdown (Fig. 5D-F). SIRT1 knockdown blocked the protective effect of MA on mitochondrial function in NMVMs and H9c2 cells (Fig. 5G-L). In addition, knockdown of SIRT1 abolished the inhibitory effect of MA on p38 and the promoting effect on STAT3, thereby reversing the anti-inflammatory and antioxidant effects of MA (Fig. 5M-P).

**Figure 6:**
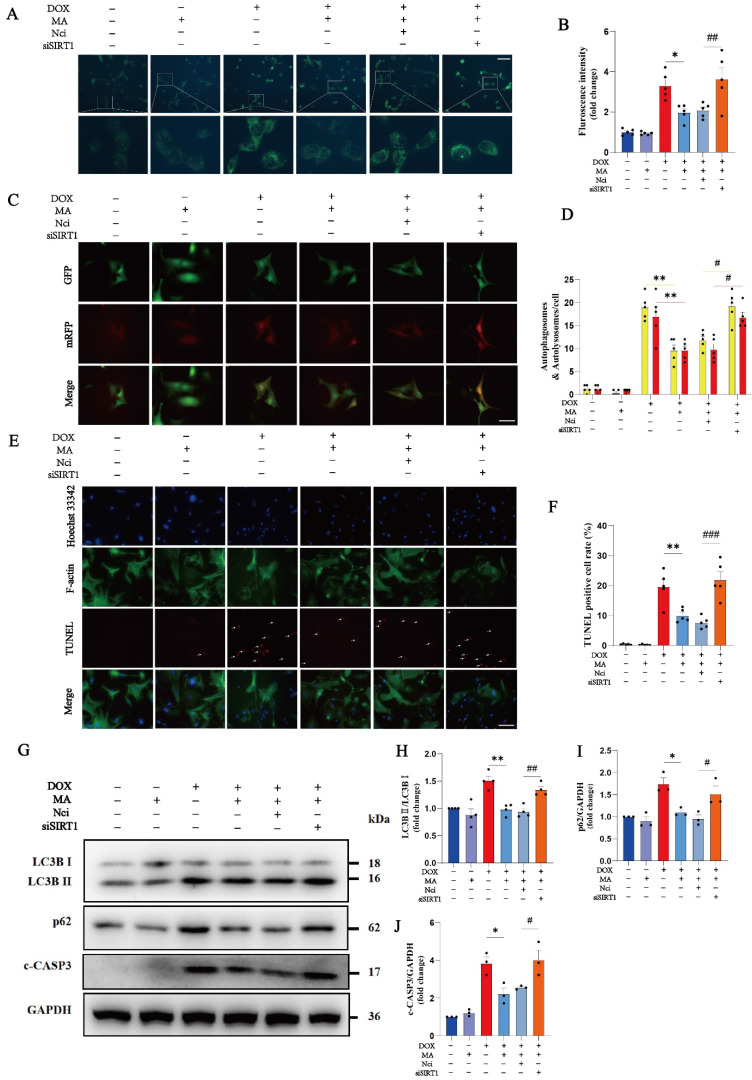
Knockdown of SIRT1 reverses the antiapoptotic and proautophagic effects of MA. (A, B) Autophagy levels in H9c2 cells were detected using an MDC assay (n=5). Scale bar: 100 µm. (C, D) NMVMs were transfected with 10 M.O.I. of tandem fluorescence-labeled LC3 adenovirus (mRFP-GFP-LC3) to detect autophagic flux. The red color represents the number of red fluorescence spots per cell, which represents the number of intracellular autolysosomes. Yellow represents the number of spots of yellow fluorescence per cell, indicating the number of intracellular autophagosomes (n=5). Scale bar: 50 µm. (E, F) TUNEL, F-actin and Hoechst 33342 staining of NMVMs (n=5). Scale bar: 100 µm. (G‒J) The expression levels of LC3B I, LC3B II, p62 and c-CASP3 were detected via Western blotting (n=3‒4). GAPDH was used as a loading control. ns: not significant, *P < 0.05, **P < 0.01, ***P < 0.001. ^#^*P* < 0.05, ^##^*P* < 0.01, and ^###^*P* < 0.001.

SIRT1 knockdown increased the percentage of autophagic vesicles, autophagic lysosomes, and TUNEL-positive cardiomyocytes, suggesting that SIRT1 plays a critical role in the protective effects of MA on DOX-induced cardiotoxicity (Fig. 6A-F). In addition, the inhibitory effects of MA on LC3B II/I, p62 and c-CASP3 levels were abolished by SIRT1 knockdown, further confirming the important role of SIRT1 in promoting autophagic flux and inhibiting cardiomyocyte apoptosis induced by MA (Fig. 6G-J).

### 3.4 MA pretreatment antagonizes DOX-induced myocardial injury and protects cardiac function *in vivo*

**Figure 7:**
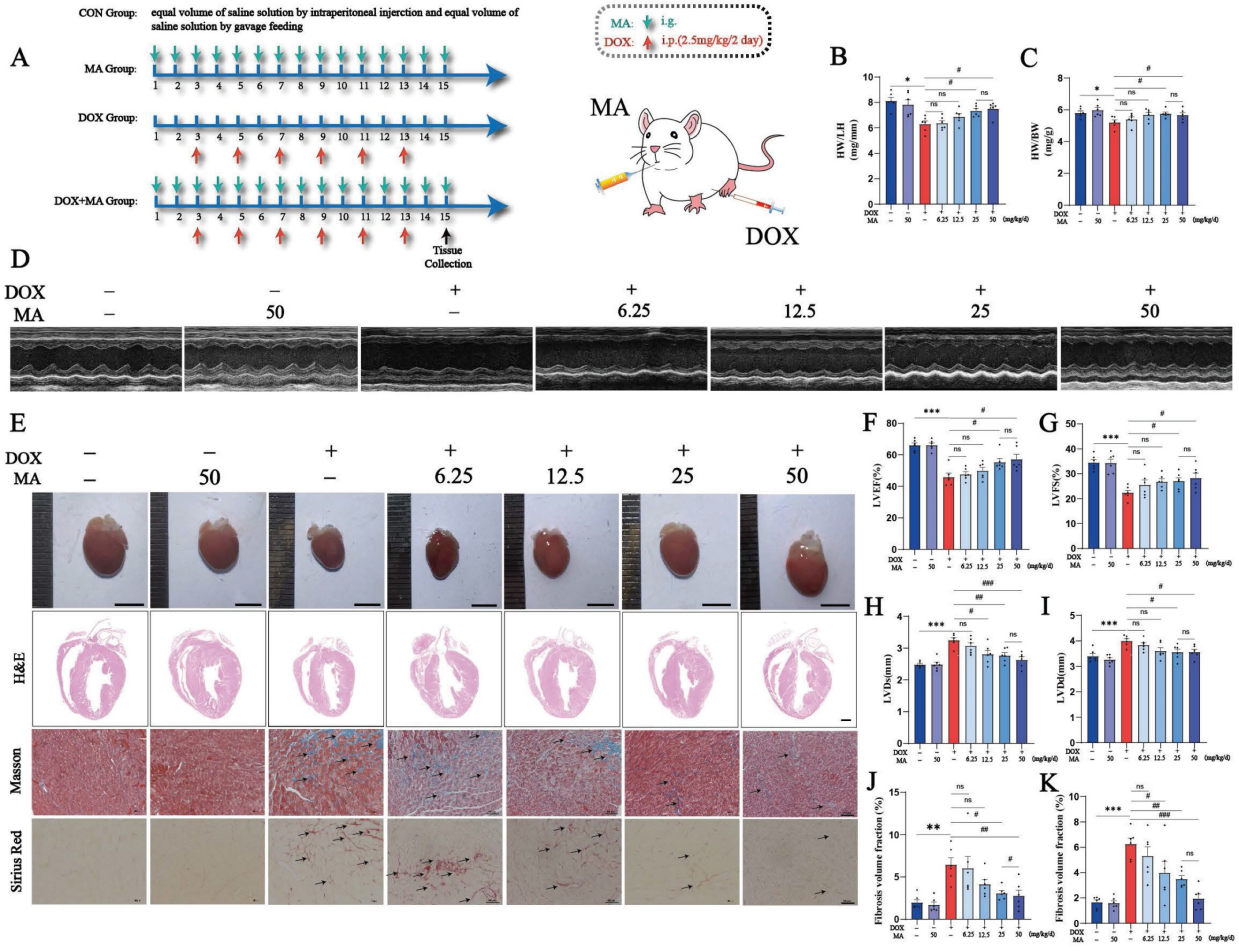
MA pretreatment inhibits cardiotoxicity caused by DOX and protects cardiac function in a mouse model of DOX injury. (A) Diagram of the animal experiment and drug intervention patterns. (B, C) Heart-to-weight ratios and heart-to-tibia length ratios of the seven groups of mice (n=6). (D, F‒I) Representative images of echocardiography and changes in LVEF, LVFS, LVIDs, and LVIDd in each group (n=6). (E) Representative images of mouse hearts (n=6, scale bar: 0.5 cm), H&E-stained samples (n=6, scale bar: 1000 µm), and Masson's trichrome- and Sirius red-stained samples (n=6, scale bar: 50 µm) from the seven groups of mouse hearts. (J) Semiquantitative analysis of the fibrotic area ratio determined using Masson's trichrome staining (n=6). (K) Semiquantitative analysis of the fibrotic area ratio determined using Sirius red staining (n=6). ns: not significant, *P < 0.05, **P < 0.01, and ***P < 0.001 compared with the control group. ^#^*P* < 0.05, ^##^*P* < 0.01, and ^###^*P* < 0.001 compared with the DOX group.

A mouse model of heart failure was established via the intraperitoneal injection of DOX to investigate the cardioprotective effects of MA *in vivo* (Fig. 7A). The mice were injected intraperitoneally with a low dose of DOX (2.5 mg/kg/day) every two days for a total of six injections, with a cumulative dose of 15 mg/kg, to establish a DOX-induced myocardial injury model in mice and to simulate the process of chemotherapy in clinical patients. Moreover, MA (6.25, 12.5, 25, or 50 mg/kg/day) was administered daily by gavage starting 2 days before the DOX injection. The echocardiographic results revealed that the LVEF and LVFS were significantly lower in the DOX group than in the control group, whereas the LVIDd and LVIDs were greater in the DOX group. Interestingly, the decreases in LVEF and LVFS and the increases in LVIDd and LVIDs were attenuated in MA-pretreated mice in a dose-dependent manner compared with those in the DOX group (Fig. 7D, F-I).

Compared with control mice, DOX-treated mice had smaller, abnormally shaped and lighter heart weights; the decrease in heart weight was attenuated, and heart morphology improved in a dose-dependent manner after MA pretreatment (Fig. 7B, C, E). MA improves DOX-induced heart tissue damage and myocardial fibrosis. H&E staining revealed that the hearts of the CON and MA groups had a normal morphology with well-arranged cardiomyocytes and had no abnormalities, such as hemorrhage, inflammatory cell infiltration or edema. In contrast, the DOX group exhibited overall cardiac shrinkage, thinning of the ventricular wall and septum, structural disorganization of cardiomyocytes, cellular edema, and inflammatory cell infiltration. Interestingly, MA pretreatment improved the cardiac morphology and reduced edema and inflammation in cardiomyocytes. Masson's trichrome staining also revealed that MA pretreatment attenuated DOX-induced myocardial fibrosis (Fig. 7E, J, K). The cardioprotective effect of MA was dose dependent, with doses of 25 mg/kg/day and 50 mg/kg/day having the best cardioprotective effect. Therefore, we chose the 25 mg/kg/day dose for the subsequent experiments.

**Figure 8:**
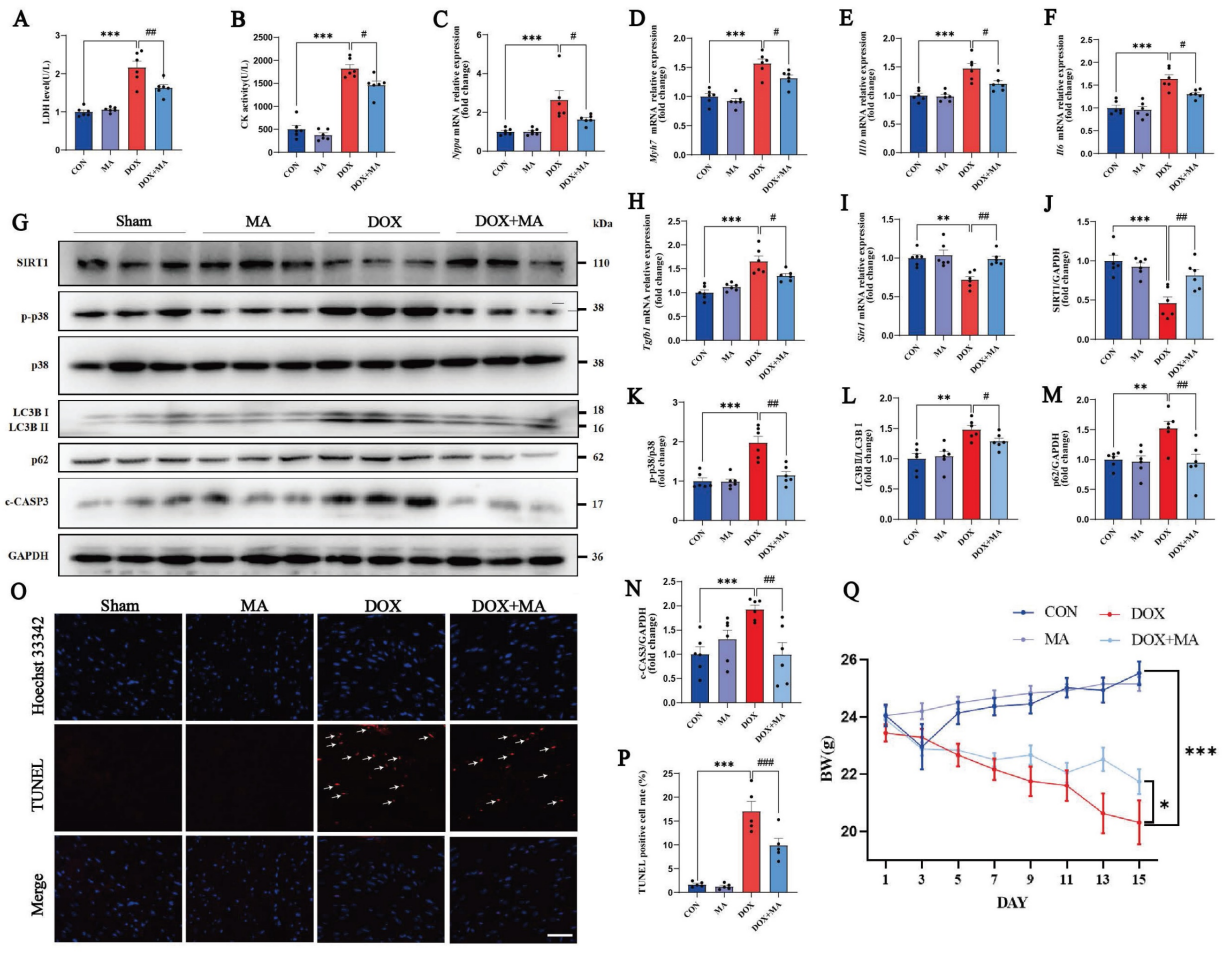
MA pretreatment attenuated DOX-induced myocardial fibrosis and apoptosis *in vivo*. (A) Measurement of LDH activity in the four groups of mice (n=6). (B) Measurement of CK activity in the four groups of mice (n=6). (C‒F, H, I) Real-time PCR was performed to detect* Nppa*, *Myh7*, *Il1b*, *Il6, Tgfb1* and *Sirt1* expression in myocardial samples from each group. (G, J‒N) The expression of SIRT1, phosphorylated and total p38, LC3B I, LC3B II, p62 and c-CASP3 was detected by Western blotting (n=3). GAPDH was used as a loading control. (O, P) TUNEL and Hoechst 33342 staining (n=5). Scale bar: 100 µm. (Q) Statistical graph of weight changes in different groups. ns: not significant, *P < 0.05, **P < 0.01, and ***P < 0.001 compared with the control group. ^#^*P* < 0.05, ^##^*P* < 0.01, and ^###^*P* < 0.001 compared with the DOX group.

In addition, at a dose of 25 mg/kg/day, MA inhibited the DOX-induced increases in the levels of the myocardial injury marker enzymes CK and LDH (Fig. 8A and B). Moreover, the expression of the embryonic genes *Nppa* and *Myh7*, which are markers of heart failure, was also significantly increased in the DOX group, as detected by real-time PCR (Fig. 8C, D). The expression of the inflammatory factors *Il1b*, *Il6*, and p38 and the myocardial fibrosis marker gene *Tgfb1* was significantly increased (Fig. 8E-H, K). On the other hand, the expression of SIRT1 was significantly decreased (Fig. 8G, I, J). The increased expression of the autophagy markers LC3B II/Ⅰ and p62 indicated impaired autophagic flux in cardiomyocytes (Fig. 8G, L, M). c-CASP3 and TUNEL positivity were both increased, indicating increased apoptosis in cardiomyocytes (Fig. 8G, N-P). The administration of MA at 25 mg/kg/day also attenuated DOX-induced weight loss in the mice (Fig. 8Q). Thus, the *in vivo* studies demonstrate that MA attenuates DOX-induced cardiotoxicity, inhibits myocardial remodeling and has a significant protective effect on cardiac function.

### 3.5 MA inhibits tumor cell proliferation, migration and invasion and enhances the anticancer effect of DOX

Interestingly, we found that MA not only attenuated DOX-induced cardiotoxicity, but also enhanced the anticancer effects of DOX by inhibiting tumor cell proliferation, migration and invasion. MA can inhibit colon cancer growth by inducing apoptosis and immunomodulation^25^. However, the antitumor mechanism of MA is not fully understood.

**Figure 9:**
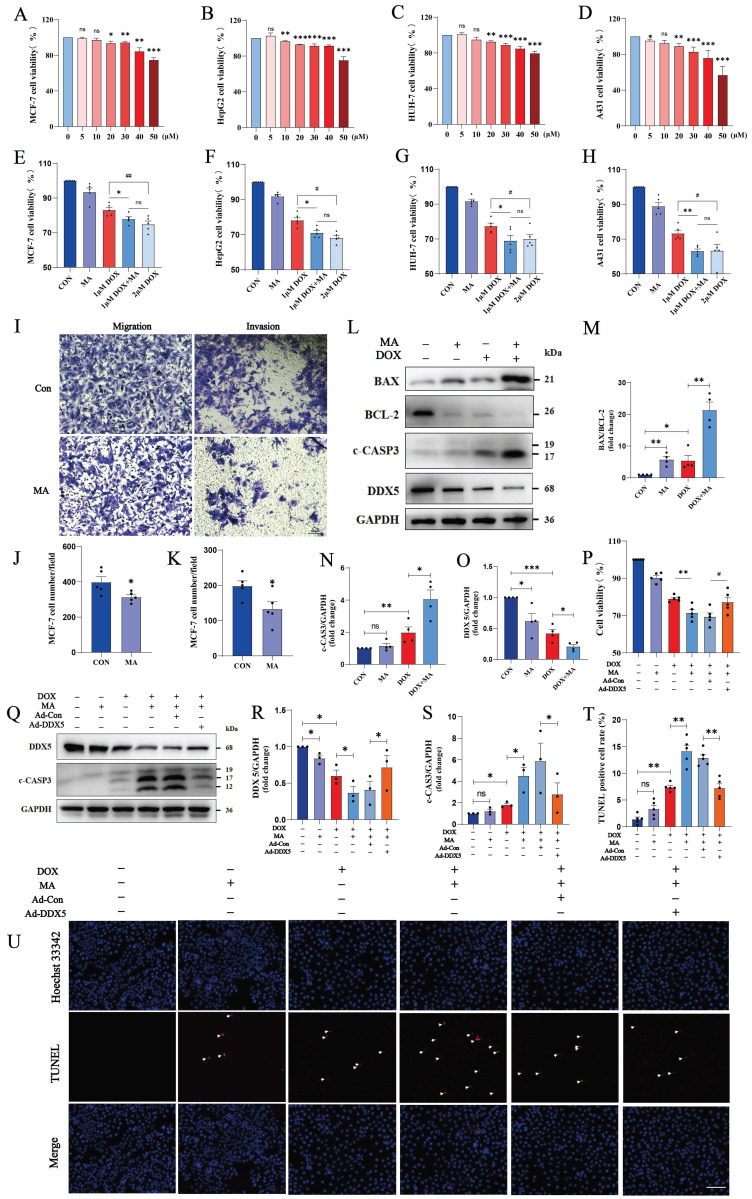
MA inhibits tumor cell proliferation, migration and invasion and enhances the anticancer effect of DOX. (A‒H) MCF-7, HepG2, Huh-7, and A431 cells were treated with 0-50 µM MA or DOX for 24 h, and cell proliferation was detected with a CCK-8 assay (n=5). (I‒K) The migration and invasive abilities of MCF-7 cells were decreased by 20 µM MA treatment (n=5). Scale bar: 50 µm. (L‒O) The expression levels of BAX, BCL-2, c-CASP3 and DDX5 in MCF-7 cells subjected to different treatments were detected by Western blotting (n=3). GAPDH was used as a loading control. (P) The viability of MCF-7 cells was detected using a CCK-8 assay (n=5). (Q‒S) The expression levels of DDX5 and c-CASP3 in MCF-7 cells subjected to different treatments were detected using Western blotting (n=3). GAPDH was used as a loading control. (T, U) TUNEL and Hoechst 33342 staining of MCF-7 cells treated with 20 µM MA and/or 1 µM DOX (n=5). Scale bar: 100 µm. ns: not significant, *P < 0.05, **P < 0.01, ***P < 0.001. ^#^*P* < 0.05, ^##^*P* < 0.01, and ^###^*P* < 0.001.

We found that 20 µM MA inhibited the proliferation of many cancer cell lines, such as MCF-7, HepG2, Huh-7 and A431 cells (Fig. 9A-D). Next, we treated tumor cells with MA in combination with DOX to explore the anticancer effect of MA on tumor cells. Compared with DOX alone, the combination therapy of MA and DOX significantly inhibited tumor cell proliferation, and the same antitumor effect was achieved with a reduced DOX dose. Thus, the combination of MA and DOX could reduce not only the dose of DOX but also the side effects of DOX on cardiomyocytes caused (Fig. 9E-H, Fig. S1A-D). Moreover, we found that 20 µM MA inhibited the migration and invasion of MCF-7, HepG2, Huh-7 and A431 cells (Fig. 9I-K, Fig. S1E-H).

TUNEL staining revealed that apoptosis was not significant when MCF-7 cells were treated with 20 µM MA alone, but apoptosis was significantly increased in the cotreatment group (20 µM MA and 1 µM DOX). Similarly, we observed the same effect on HepG2 cells (20 µM MA and 5 µM DOX) and A431 cells (20 µM MA and 3 µM DOX) (Fig. 9T, U; Fig. S1I-L). Furthermore, MA promoted increases in c-CASP3 expression and the BAX/BCL-2 ratio in MCF-7, HepG2 and A431 cells (Fig. 9L-N, Fig. S1M-O, Q-S).

The DEAD-box RNA helicase family plays important roles in cancer development and tumor metabolism, of which DDX5 is the most representative member. Increasing evidence indicates that DDX5 plays critical roles in tumorigenesis, proliferation, metastasis, progression and drug resistance^24^. MA inhibited DDX5 expression in MCF-7 cells (Fig. 9L, O) and in A431 and HepG2 cells Fig. S1N, P, R, T). We constructed a plasmid expressing human DDX5 (NM_001320595.2) using the pcDNA3.1 vector and transfected it into MCF-7 cells. The overexpression of DDX5 in MCF-7 cells resulted in a significant increase in cell viability and a decrease in c-CASP3 levels, indicating that the overexpression of DDX5 reversed the antitumor effects of DOX and MA on tumor cells and led to a significant decrease in the anticancer effect (Fig. 9P-S). Therefore, MA not only inhibits cancer cell proliferation, migration and invasive ability but can also reduce the dose of DOX and increase the anticancer effect. These findings suggest that MA can be used as an anticancer adjuvant or chemosensitizing agent, which can be applied in clinical tumor chemotherapy.

## 4. Discussion

As some of the most widely used chemotherapeutic agents, anthracyclines are known for their broad anticancer spectrum, potent anticancer activity, and remarkable therapeutic efficacy(31. However, anthracycline-induced cardiotoxic effects limit their clinical use^32^. Therefore, finding a cardioprotective drug for coadministration with DOX is crucial. In this study, we found that MA, a pentacyclic triterpenoid, not only attenuated DOX-induced cardiotoxic effects through the SIRT1-activated signaling pathway but also enhanced the anticancer effects of DOX, providing a dual beneficial effect on tumor therapy.

*C. asiatica* is a triterpene-rich herb commonly used to treat dermatologic, metabolic, neurologic, and cardiovascular disorders^33^. The main active constituents of *C. asiatica* are pentacyclic triterpenoids, including asiatic acid, asiaticoside, MA, and madecassoside^33^, ^34^. Previous studies have shown that *C. asiatica* exerts anti-inflammatory, antioxidant and neuroprotective effects in both vivo and *in vitro*^33^, ^34^. In recent years, an increasing number of studies have shown that *C. asiatica* also has potential for the treatment of cardiovascular diseases. Studies on cardiovascular disease have shown that various extracts and compounds isolated from *C. asiatica* can reduce symptoms of cardiac hypertrophy, myocardial ischemia, atherosclerosis, hypertension, hyperlipidemia, hyperglycemia, diabetes mellitus, oxidative stress and inflammation^12^. Among the triterpenes in *C. asiatica*, MA is a less studied triterpenoid. Notably, one study showed that oral administration of 200 mg/kg aqueous extract of *C. asiatica* reduced DOX-induced elevations in serum LDH, creatine phosphokinase, glutamic acid transaminase, and glutamic pyruvic acid transaminase levels in rats^35^. In addition, aqueous extracts of *C. asiatica* increased the activities of antioxidant enzymes such as total reduced glutathione, glutathione S-transferase, GPX, and SOD in adriamycin-induced rat heart tissues^35^. These studies suggest that coumaric acid may have cardioprotective effects. Therefore, searching for compounds in *C. asiatica* that can inhibit DOX-induced cardiotoxicity is important. In this study, we found that MA not only attenuates DOX-induced myocardial toxicity, oxidative stress and inflammation, but also exerts a beneficial cardioprotective effect by reducing autophagic flux blockage and cardiomyocyte apoptosis. Mechanistically, MA can bind to SIRT1, promote SIRT1 activation and upregulate SIRT1 expression, thus attenuating DOX-induced cardiotoxicity *in vivo* and *in vitro*.

As the world becomes an aging society, the number of cancer patients continues to rise due to a variety of factors including global aging, environmental pollution, poor diet and stress^36^. Although the development of modern medicine has led to the development of more chemotherapy drugs with fewer side effects, anthracycline-based chemotherapy drugs are still the most widely used cancer drugs in clinical chemotherapy^37^, ^38^. The clinical use of anthracyclines is also associated with many side effects in cancer patients, of which cardiotoxicity is one of the most serious. The only drug approved by the US Food and Drug Administration (FDA) for the treatment of DOX-induced cardiotoxicity is dexrazoxane, which prevents anthracycline cardiotoxicity by acting as an iron chelator^39^, ^40^. Some patients may experience side effects such as nausea, vomiting, diarrhea, mucositis, alopecia, liver enzyme abnormalities and myelotoxicity (neutropenia, leukopenia, granulocytopenia and thrombocytopenia) after the use of dexrazoxane^41^, ^42^. In addition, dexrazoxane may reduce the sensitivity of cancer cells to chemotherapy and may also pose a long-term carcinogenic risk: the incidence of myelodysplastic syndrome and acute myeloid leukemia is three times higher in control pediatric patients treated with dexrazoxane, limiting its clinical application^41^-^43^. Therefore, the search for new therapeutic agents, especially natural compounds, with the ability to treat DOX cardiotoxicity with fewer side effects is important.

Previous studies have shown that oral administration of MA reduces the extent and incidence of tumors in a mouse model of AOM/DSS-induced colitis-associated rectal cancer^25^. MA attenuates colitis-associated colorectal cancer by inhibiting the expression of IL-17 in γδT17 cells, preventing the recruitment of myeloid-derived suppressor cells, and enhancing the antitumor immune response^25^. In our study, MA inhibited the proliferation, migration and invasion of tumor cell lines such as A431, HepG2 and Huh-7 cells. When combined with DOX, it achieved a better chemotherapeutic effect with a lower DOX dose and fewer side effects. Thus, for the first time, we found that MA has both inhibitory effects on tumor growth and counteracts the myocardial toxicity of chemotherapeutic agents, and it has great potential to attenuate cardiac chemotherapeutic injury and promote tumor chemotherapeutic efficacy. Compared with dexrazoxane, MA, which is derived naturally from *C. asiatica*, has fewer side effects and is more readily available. In addition, MA increases the sensitivity of tumors to chemotherapeutic agents and promotes the efficacy of chemotherapeutic treatment.

DDX5 plays critical roles in tumorigenesis, proliferation, metastasis and treatment resistance and is an important regulator and potential biomarker and target^23^. Therefore, targeted inhibition of DDX5 can switch cancer cells from undergoing growth arrest to apoptosis and cell death. In our study, MA inhibited DDX5 expression in HepG2 and A431 cells and enhanced the inhibitory effect of DOX on DDX5 expression in tumor cells. Taken together, these findings suggest that MA likely exerts its anticancer effects by inhibiting the expression of the key oncogenic factor DDX5 and then inducing apoptosis in cancer cells. Therefore, MA may be an anticancer and chemotherapeutic potentiator with great clinical potential and important applications in future clinical tumor therapy.

In conclusion, our study revealed that MA not only prevents DOX-induced myocardial toxicity but also enhances the anticancer effect of chemotherapeutic drugs. Due to the wide range of beneficial biological functions and pharmacological activities of MA, it can be used as a preprotective agent before anthracycline chemotherapy treatment to prevent and minimize the cardiotoxic effects of anthracycline chemotherapy and enhance its anticancer effects. However, this study has several limitations. Although we have demonstrated the cardioprotective effects of MA on cellular and mouse models, we lack clinical trials in humans to validate the effects of MA. Therefore, further in-depth clinical trials are needed to demonstrate its clinical efficacy in cancer patients.

## 5. Conclusions

In conclusion, our data suggest that MA exerts cardioprotective effects by promoting the expression and activity of SIRT1 and reducing DOX-induced autophagic flux blockade and apoptosis in cardiomyocytes. Moreover, MA inhibits cancer cell proliferation and enhances the chemotherapeutic effect of DOX. These findings suggest that MA may not only be a promising natural compound for the treatment of DOX-induced cardiotoxicity but also provide a strategy for synergistic anticancer therapy and combination therapy with anticancer drugs to prevent chemotherapy-induced cardiac damage.

## Supplementary Material

Supplementary figure and tables.

## Figures and Tables

**Scheme 1:**
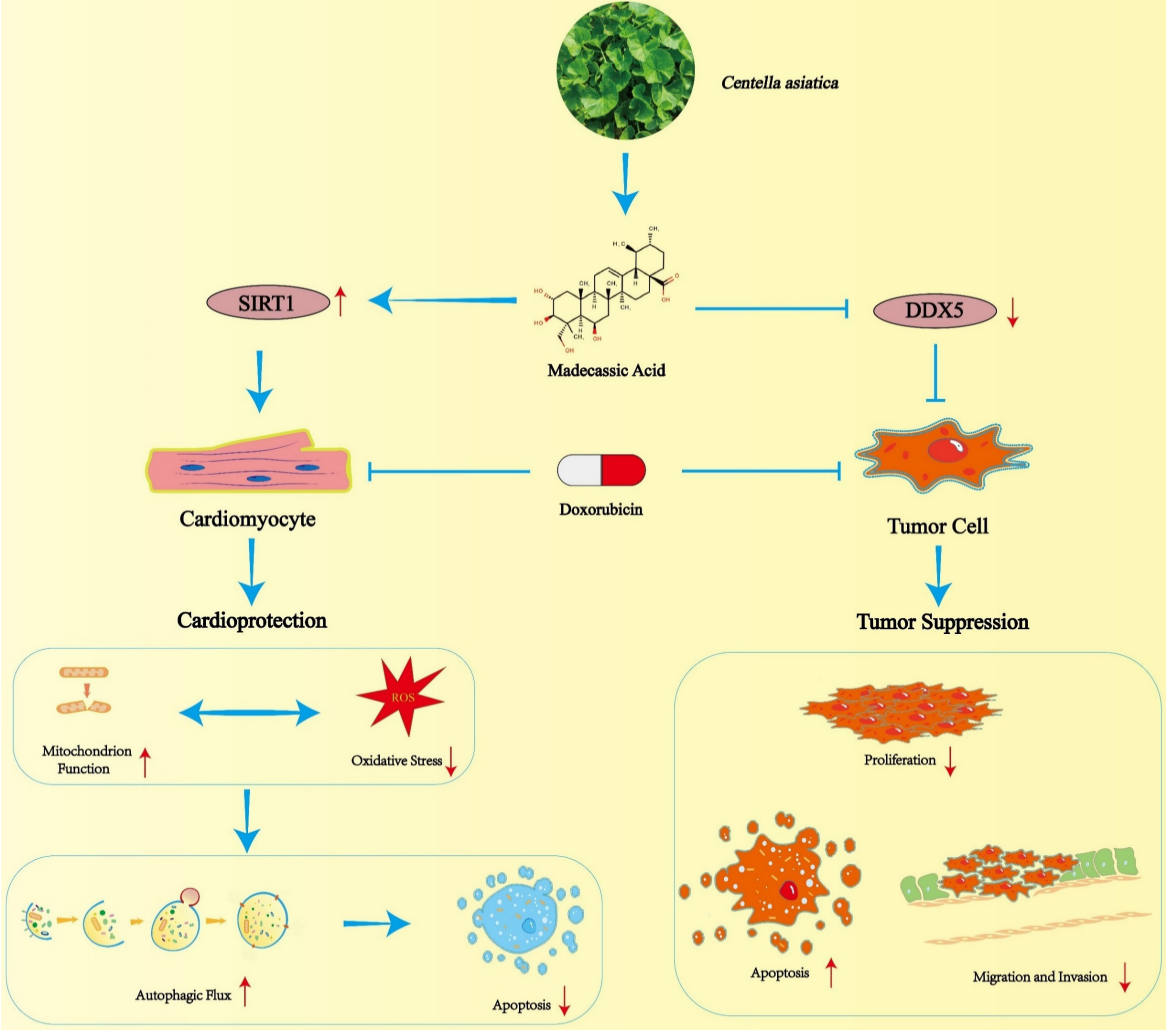
Schematic illustration of MA mediated cardioprotection and tumor suppression.
