# Supplementary material for: The dual functions of the pentacyclic triterpenoid madecassic acid in ameliorating doxorubicin-induced cardiotoxicity and enhancing the antitumor efficacy of doxorubicin
Source: Int J Biol Sci. 2024 Oct 7;20(14):5396–414. doi: 10.7150/ijbs.97418 (PMC11528453; doi:10.7150/ijbs.97418)
Supplement: Supplementary file 1 — Supplementary figure and tables. [file ijbsv20p5396s1.pdf]

Supplementary Material

Supplement figure

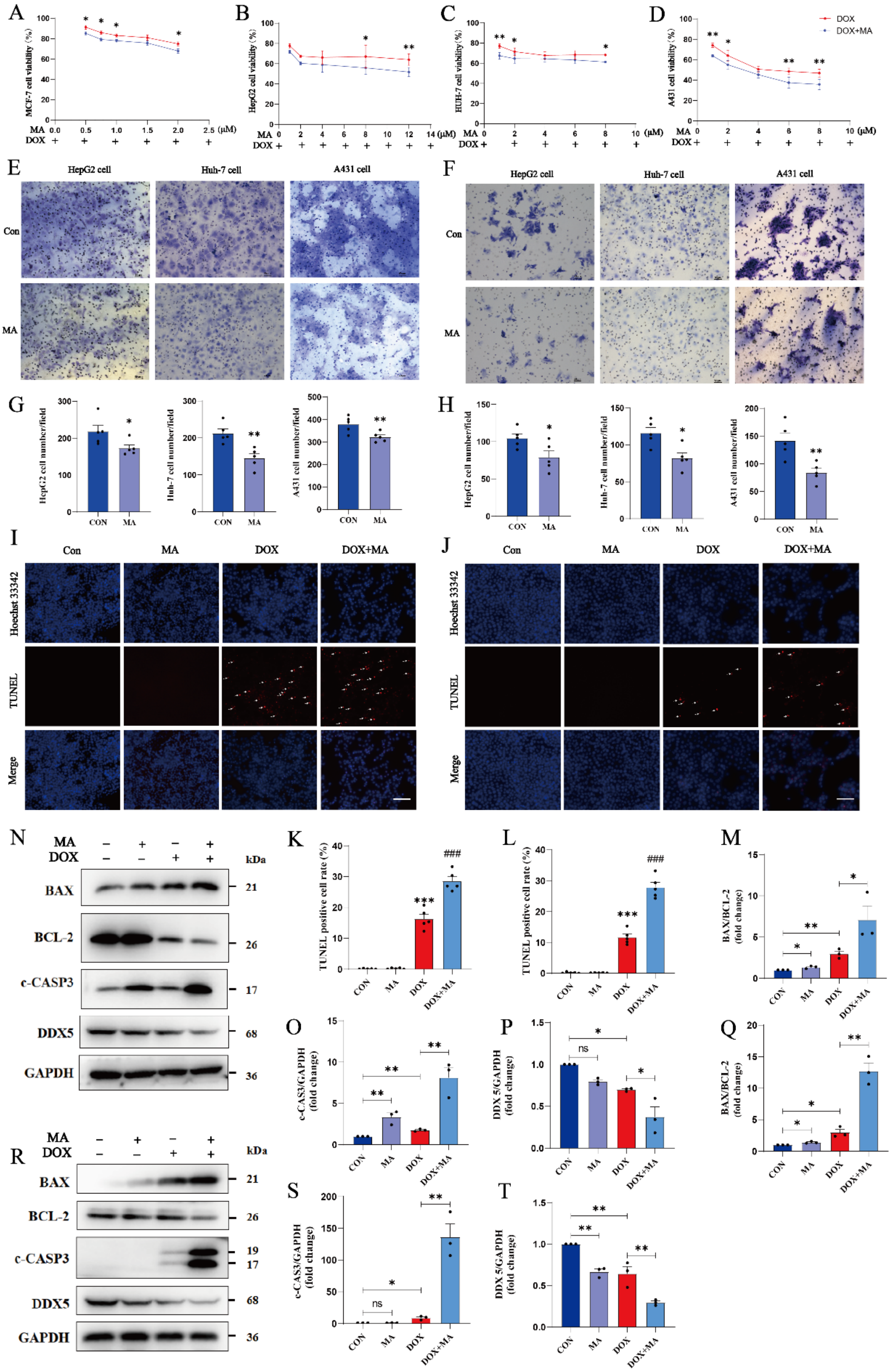

Supplement figure 1. MA inhibits tumor cell proliferation, migration and invasion and enhances the anticancer effect of DOX. (A-D) The effects of different doses of DOX and the combination of DOX and MA on the proliferative viability of MCF, Hepg2, Huh-7, and A431 cells were examined by CCK-8 (n=5). (E, G) Migration ability of Hepg2, Huh-7, and A431 cells were all decreased by 20μM MA treatment (n=5). Scale bar: 50μm. (F, H) Invasive ability of Hepg2, Huh-7, and A431 cells were all decreased by 20μM MA treatment (n=5). Scale bar: 50μm. (I, K) TUNEL and Hoechst 33342 staining of HepG2 cells treated with 20μM MA (n=5). Scale bar: 100μm. (J, L) TUNEL and Hoechst 33342 staining of A431 cells treated with 20μM MA (n=5). Scale bar: 100μm. (M-P) Expression levels of BAX, BCL-2, c-CASP3 and DDX5 were detected by Western blot in A431 cells under different treatments (n=3). (Q-T) Expression levels of BAX, BCL-2, c-CASP3, DDX5 were detected by Western blot in HepG2 cells under different treatments (n=3). GAPDH was used as a loading control. ns: not significant, $^{*}P < 0.05$, $^{**}P < 0.01$, $^{***}P < 0.001$ vs. control group. $^{\#}P < 0.05$, $^{\#\#}P < 0.01$, $^{\#\#\#}P < 0.001$ vs. DOX group.

Supplement table

Supplement table 1. List of Abbreviations

Acronym	Full name
MA	madecassic acid
DOX	doxorubicin
SIRT1	sirtuin 1
miRNA	microRNA
lncRNA	long non-coding RNA
DDX5	DEAD-box helicase 5
NAD^+	nicotinamide adenosine dinucleotide
LDH	lactate dehydrogenase
CK	creatine kinase
NMCMs	neonatal mouse ventricular myocytes

ROS	reactive oxygen species
Nppa	natriuretic peptide A
Myh7	myosin heavy chain 7
TGF-β	transforming growth factor-β
SQSTM1/p62	sequestosome 1
LC3B	microtubule associated protein 1 light chain 3 beta
p38	*p38 MAP kinase*
STAT3	signal transducer and activator of transcription 3
c-CAS3	*cleaved Caspase-3*
LVEF	*left ventricular ejection fraction*
LVFS	left Ventricular Fraction Shortening
LVIDd	left ventricular internal diameter diastolic
LVIDs	left ventricular internal diameter systolic
BAX	BCL2-Associated X
BCL-2	B-cell lymphoma-2
GAPDH	glyceraldehyde-3-phosphate dehydrogenase

Supplement table 2. List of antibodies

Antibodies	Manufacturer	Cat. No.	Source	Application(s) and Dilution
BAX	Cell Signaling Technology	2772	rabbit	WB (1:1000)
BCL2	Cell Signaling Technology	3498	rabbit	WB (1:1000)
Acetylated-Lysine	Cell Signaling Technology	9441	rabbit	WB (1:1000)
cleaved-Caspase-3	Cell Signaling Technology	9661	rabbit	WB (1:1000)
DDX5	Cell Signaling Technology	9877	rabbit	WB (1:1000)
Phospho-STAT3 （Tyr705）	Cell Signaling Technology	9145	rabbit	WB (1:1000)
STAT3	Cell Signaling Technology	4904	Rabbit	WB (1:1000)
Phosphor-p44/42 MAPK				

(ERK1/2) (Thr202/Tyr204)	Cell Signaling Technology	4370	rabbit	WB (1:1000)
p44/42 MAPK	Cell Signaling Technology	4695	rabbit	WB (1:1000)
GAPDH	Cell Signaling Technology	2118	rabbit	WB (1:1000)
LC3B	Sigma-Aldrich	SAB5701328	rabbit	WB (1:1000)
Phospho-SAPK/JNK (Thr183/Tyr185)	Cell Signaling Technology	9251	rabbit	WB (1:1000)
SAPK/JNK	Cell Signaling Technology	9252	rabbit	WB (1:1000)
p62	Cell Signaling Technology	5114	rabbit	WB (1:1000)
Phosphor- p38 MAPK (Thr180/Tyr182)	Cell Signaling Technology	9215	rabbit	WB (1:1000)
p38 MAPK	Cell Signaling Technology	8690	rabbit	WB (1:1000)
SIRT1	Cell Signaling Technology	9475	rabbit	WB (1:1000)
Anti-rabbit IgG	Cell Signaling Technology	7074	goat	WB (1:1000)
